# Esophageal Gastrointestinal Stromal Tumor: Diagnostic Complexity and Management Pitfalls

**DOI:** 10.1155/2013/968394

**Published:** 2013-04-30

**Authors:** Charalampos G. Markakis, Eleftherios D. Spartalis, Emmanouil Liarmakopoulos, Evangelia G. Kavoura, Periklis Tomos

**Affiliations:** ^1^Second Department of Propedeutic Surgery, University of Athens, Medical School, “Laiko” General Hospital, Agiou Thoma 17, 11527 Athens, Greece; ^2^First Department of Pathology, University of Athens, Medical School, 11527 Athens, Greece

## Abstract

*Introduction*. Gastrointestinal stromal tumors of the esophagus are rare. *Case Presentation*. This is a case of a 50-year-old male patient who was referred to our department complaining of atypical chest pain. A chest computed tomographic scan and endoscopic ultrasound revealed a submucosal esophageal tumor measuring 5 cm in its largest diameter. Suspecting a leiomyoma, we performed enucleation via right thoracotomy. The pathology report yielded a diagnosis of an esophageal gastrointestinal stromal tumor. The patient has shown no evidence of recurrence one year postoperatively. *Conclusions*. This report illustrates the complexity and dilemmas inherent in diagnosing and treating esophageal GISTs.

## 1. Introduction

Gastrointestinal stromal tumors (GISTs) are the most common mesenchymal neoplasms of the gastrointestinal tract [1]. After the discovery of c-kit mutations, their accurate diagnosis and differentiation from other mesenchymal tumors became possible and the use of imatinib mesylate provided new therapeutic options. Consequently, there was interest in these tumors and their management and prognosis has been extensively investigated and standardized.

Esophageal GISTs, in contrast, are rare, amounting to 12.7–28% of mesenchymal esophageal tumors or 2% of all GISTs [1–3] and their diagnosis and management are still challenging, as illustrated in the following case.

## 2. Case Presentation

**Figure 1:**
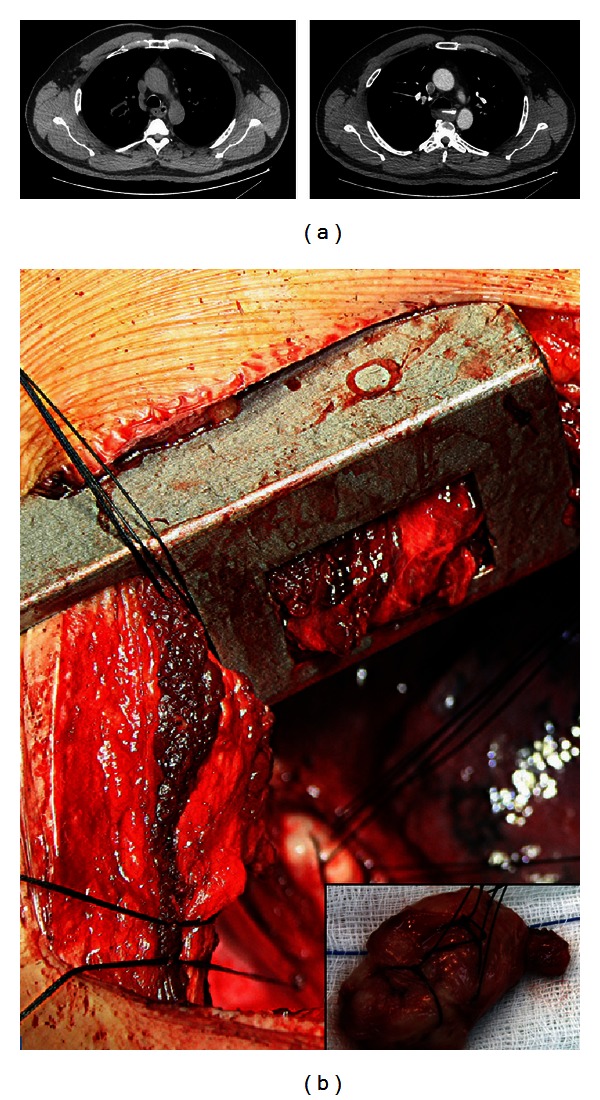
(a) CT scan of the chest, showing a well-circumscribed 5 cm submucosal esophageal mass. (b) Intraoperative view of the tumor (inset: macroscopic view of the resected specimen).

A 50 year old Caucasian male was referred to the thoracic surgery department for evaluation of an intramural esophageal mass. The patient complained of atypical chest pain of gradual onset over the previous 6 months. He denied weight loss, dysphagia, upper GI bleeding, reflux, or other symptoms. The patient's medical history included hypertension and a 30-pack-year smoking history. After a chest radiograph failed to show any pathology, a computed tomography (CT) scan was ordered which revealed showed a 5 cm mass on the midesophagus at the junction of the azygos vein with the superior vena cava (Figure 1). Endoscopy showed a normal esophageal mucosa and endoscopic ultrasound a smooth, submucosal mass. A CT scan of the abdomen did not show any evidence of distant metastases. 

The mass was approached via a right posterolateral thoracotomy (Figure 1). The subcarinal lymph nodes were found to be enlarged and were sent for frozen section, which was negative for malignancy. The mass was enucleated from the esophageal wall by gently detaching it from the mucosa. No adhesions with the mucosa or muscularis were noted, and the mass was excised with its capsule intact. A frozen section of the mass indicated the mesenchymal origin, with a possible diagnosis of leiomyoma. The muscular layer of the esophagus was repaired with vicryl 4-0 interrupted sutures and covered with parietal pleura. Integrity of the esophageal mucosa was established by intraoperative endoscopy. An upper gastrointestinal series on postoperative day 1 showed no evidence of a leak, and the patient was uneventfully discharged on the 6th postoperative day.

**Figure 2:**
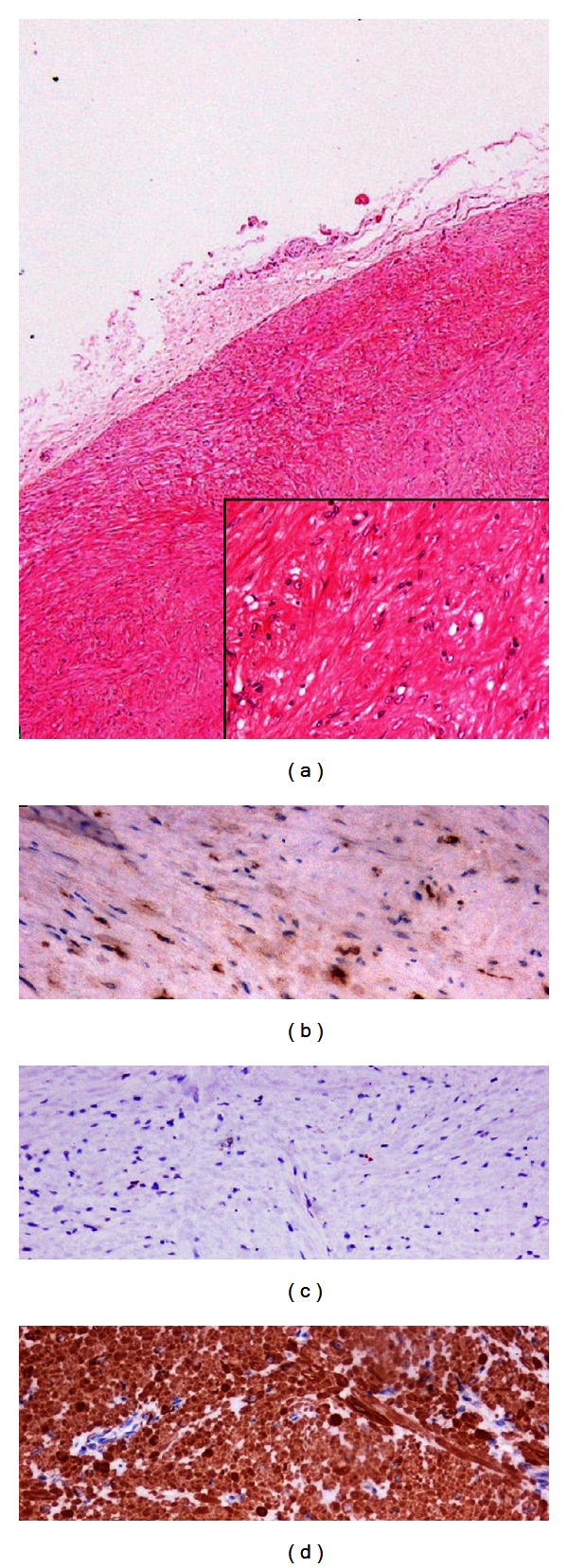
Microscopic view of the tumor. (a) H/E ×40 (inset H/E X200), (b) c-kit ×200, (c) Ki-67 ×200, and (d) SMA (a smooth muscle actin) ×200.

On macroscopic examination, the mass was 5.5 × 3.5 × 1.5 cm in size and grayish in color with a fasciated texture (Figure 1). Histologically the mass corresponded to an encapsulated mesenchymatous neoplasm, consisting of fibrous and muscle fascicles with sparse round and spindle cells (Figure 2(a)). No neoplastic cells were found to infiltrate the margins of the capsule. There was no evidence of necrosis and <2 mitoses per 50 high-power fields. Less than 1% of cells stained positive for Ki67. The diagnosis of a GIST was established by immunohistochemistry, which revealed a positive immunoreaction to c-kit and CD34 (Figures 2(b) and 2(c)). There was also an unusual positive reaction to smooth muscle actin (SMA) (Figure 2(d)) [3, 4]. All excised lymph nodes were negative.

After a multidisciplinary meeting the patient received adjuvant therapy (imatinib mesylate 400 mg/d for 1 year). He is closely followedup with endoscopy and CT scans every 3 months and is currently free of disease one year after surgery.

## 3. Discussion

Esophageal GISTs are difficult to diagnose preoperatively since there are no specific findings to differentiate them from far more common leiomyomas when their clinical presentation, endoscopy, endoscopic ultrasound, or CT scan is reviewed [1, 4, 5]. Both GISTs and leiomyomas are hypoechoic lesions originating from the muscularis propria or muscularis mucosa on endoscopic ultrasound, while lipomas are hyperechoic and can be easily differentiated [6]. Definitive diagnosis can be made by fine-needle aspiration but is not usually performed for esophageal submucosal lesions. This is due to the concern that it may spread malignant disease or induce scarring that might make safe enucleation impossible [1, 3]. Blum et al. recommended biopsy for tumors larger than 2 cm, enlarging tumors, or tumors positive on PET scan. In their series they encountered adhesions to the mucosa or muscularis in all GISTs (but not in 2 leiomyomas) after biopsy, in contrast to other reports [1, 2, 5]. A selective approach to biopsy based on tumor size (>5 cm) and suspicious radiological appearance is warranted until the risks of biopsy compared to the benefit of accurate preoperative diagnosis and planning are determined. As stated above, we did not perform a preoperative biopsy in our patient, while a PET scan might have been appropriate, but it was not possible to obtain it with the patient's insurance in our institution. Immunohistochemical staining can differentiate GISTs, which have c-kit mutations and are positive for CD117 and CD34 from leiomyomas, which are CD34 and CD117 negative, with no c-kit mutations. Leiomyomas are also positive for desmin and smooth muscle actin, while GISTs are usually (but not always) negative [4].

Another contentious point is the type of surgery indicated for esophageal GISTs. While the recommendation for GISTs found in other locations is a wide local excision, the increased morbidity of esophageal resection has to be taken into account. Conflicting data exist in the literature; while some authors report poor results with mortality of up to 59% [3, 4], it is difficult to attribute these to the extent of resection, as other studies such as that of Lee et al. reporting no recurrences in 5 GISTs treated by enucleation [1]. With no strong evidence available, the recommendations from the small existing case series are contradictory to suggested approaches ranging from esophagectomy to endoscopic enucleation [1, 2, 4]. The National Institute of Health (NIH) risk stratification criteria as well as other risk factors described subsequently can be used [7, 8]. There appears to be a poor prognosis in patients with tumors >9 cm and the opposite is true for tumors <5 cm [3, 4]. Our patient had several favorable prognostic factors, namely, low mitotic count, no necrosis, low percentage of Ki-67 positive cells, and negative lymph nodes. Furthermore, the tumor had a clearly defined capsule which was not breached and the tumor margins were clear. On the other hand, the size of the patient's tumor and its localization in the esophagus are reasons to consider enucleation a possibly risky strategy. We felt that we had to offer this patient, who was of low surgical risk, formal resection of the tumor site, which he refused. Concern over a possibly inadequate resection led us to administer adjuvant therapy, which has been shown to result in increased recurrence-free survival in large tumors at high risk of recurrence in the ACOSOG Z9001 trial [9].

## 4. Conclusions

This case illustrates the complexity and dilemmas in diagnosing and treating esophageal GISTs. To accumulate high-level evidence sufficient to support specific guidelines, a multi-institutional study or even an international registry of such lesions is needed. Until then, each patient should be evaluated individually by surgical risk, tumor size and biology and should actively participate in the management decision process.

## References

[1] Lee HJ, Park SI, Kim DK, Kim YH (2009). Surgical resection of esophageal gastrointestinal stromal tumors. *The Annals of Thoracic Surgery*.

[2] Von Rahden BHA, Stein HJ, Feussner H, Siewert JR (2004). Enucleation of submucosal tumors of the esophagus: minimally invasive versus open approach. *Surgical Endoscopy and Other Interventional Techniques*.

[3] Jiang P, Jiao Z, Han B (2010). Clinical characteristics and surgical treatment of oesophageal gastrointestinal stromal tumours. *European Journal of Cardio-thoracic Surgery*.

[4] Miettinen M, Sarlomo-Rikala M, Sobin LH, Lasota J (2000). Esophageal stromal tumors: a clinicopathologic, immunohistochemical, and molecular genetic study of 17 cases and comparison with esophageal leiomyomas and leiomyosarcomas. *The American Journal of Surgical Pathology*.

[5] Blum MG, Bilimoria KY, Wayne JD, de Hoyos AL, Talamonti MS, Adley B (2007). Surgical considerations for the management and resection of esophageal gastrointestinal stromal tumors. *The Annals of Thoracic Surgery*.

[6] Bhatia V, Tajika M, Rastogi A (2010). Upper gastrointestinal submucosal lesions—clinical and endosonographic evaluation and management. *Tropical gastroenterology*.

[7] Fletcher CDM, Berman JJ, Corless C (2002). Diagnosis of gastrointestinal stromal tumors: a consensus approach. *Human Pathology*.

[8] Rutkowski P, Nowecki ZI, Michej W (2007). Risk criteria and prognostic factors for predicting recurrences after resection of primary gastrointestinal stromal tumor. *The Annals of Surgical Oncology*.

[9] Dematteo RP, Ballman KV, Antonescu CR (2009). Adjuvant imatinib mesylate after resection of localised, primary gastrointestinal stromal tumour: a randomised, double-blind, placebo-controlled trial. *The Lancet*.

